# Safety of growth hormone replacement in survivors of cancer and intracranial and pituitary tumours: a consensus statement

**DOI:** 10.1530/EJE-21-1186

**Published:** 2022-03-23

**Authors:** Margaret C S Boguszewski, Cesar L Boguszewski, Wassim Chemaililly, Laurie E Cohen, Judith Gebauer, Claire Higham, Andrew R Hoffman, Michel Polak, Kevin C J Yuen, Nathalie Alos, Zoltan Antal, Martin Bidlingmaier, Beverley M K Biller, George Brabant, Catherine S Y Choong, Stefano Cianfarani, Peter E Clayton, Regis Coutant, Adriane A Cardoso-Demartini, Alberto Fernandez, Adda Grimberg, Kolbeinn Guðmundsson, Jaime Guevara-Aguirre, Ken K Y Ho, Reiko Horikawa, Andrea M Isidori, Jens Otto Lunde Jørgensen, Peter Kamenicky, Niki Karavitaki, John J Kopchick, Maya Lodish, Xiaoping Luo, Ann I McCormack, Lillian Meacham, Shlomo Melmed, Sogol Mostoufi Moab, Hermann L Müller, Sebastian J C M M Neggers, Manoel H Aguiar Oliveira, Keiichi Ozono, Patricia A Pennisi, Vera Popovic, Sally Radovick, Lars Savendahl, Philippe Touraine, Hanneke M van Santen, Gudmundur Johannsson

**Affiliations:** 1 Department of Pediatrics, Federal University of Paraná, Curitiba, Brazil; 2 SEMPR (Endocrine Division), Department of Internal Medicine, Federal University of Parana, Curitiba, Brazil; 3 Division of Endocrinology, Department of Pediatrics, University of Pittsburgh School of Medicine, Pittsburgh, Pennsylvania, USA; 4 Division of Endocrinology and Diabetes, Department of Pediatrics, The Children’s Hospital at Montefiore, Albert Einstein College of Medicine, New York, New York, USA; 5 Department of Internal Medicine I, University Medical Center Schleswig-Holstein, Luebeck, Germany; 6 Department of Endocrinology, Christie Hospital NHS Foundation Trust, University of Manchester, and Manchester Academic Health Science Centre, Manchester, UK; 7 Stanford University School of Medicine, Stanford, California, USA; 8 Department of Pediatric Endocrinology, Gynecology and Diabetology, Hôpital Universitaire Necker Enfants Malades, AP-HP, Université de Paris, Paris, France; 9 Barrow Pituitary Center, Barrow Neurological Institute, Phoenix, Arizona, USA; 10 Department of Neuroendocrinology, St. Joseph’s Hospital and Medical Center, University of Arizona College of Medicine and Creighton School of Medicine, Phoenix, Arizona, USA; 11 Division of Endocrinology, Sainte-Justine University Hospital Centre, University of Montreal, Montreal, Quebec, Canada; 12 Memorial Sloan-Kettering Cancer Center and Weill Cornel Medicine New York Presbyterian Hospital, New York, New York, USA; 13 Medizinische Klinik und Poliklinik IV, LMU Klinikum, Munich, Germany; 14 Neuroendocrine & Pituitary Tumor Clinical Center, Massachusetts General Hospital, Boston, Massachusetts, USA; 15 Department of Diabetes, Endocrinology and Gastroenterology, School of Medical Sciences, University of Manchester, Manchester, UK; 16 Department of Endocrinology and Diabetes, Perth Children’s Hospital, Child & Adolescent Health Service, Perth, Australia; 17 Division of Paediatrics, Faculty of Health & Medical Sciences, University of Western Australia, Perth, Australia; 18 Department of Systems Medicine, University of Rome Tor Vergata, Rome Italy; 19 Dipartimento Pediatrico Universitario Ospedaliero, IRCCS ‘Bambino Gesu’ Children’s Hospital, Rome Italy; 20 Department of Women’s and Children’s Health, Karolinska Institute and University Hospital, Stockholm, Sweden; 21 Faculty of Biology, Medicine & Health, University of Manchester, Manchester, UK; 22 Department of Pediatric Endocrinology, University Hospital, Angers, France; 23 Pediatric Endocrinology Unit, Department of Pediatrics, Hospital de Clínicas, Federal University of Parana, Curitiba, Brazil; 24 Endocrinology Department, Hospital Universitario de Mostoles, Mostoles, Spain; 25 Department of Pediatrics, Perelman School of Medicine, University of Pennsylvania, Philadelphia, Pennsylvania, USA; 26 Division of Endocrinology and Diabetes, Children’s Hospital of Philadelphia, Philadelphia, Pennsylvania, USA; 27 Children’s Medical Center, Landspitali – The National University Hospital of Iceland, Reykjavik, Iceland; 28 Department of Diabetes and Endocrinology, College of Medicine, Universidad San Francisco de Quito at Quito, Quito, Ecuador; 29 The Garvan Institute of Medical Research and St. Vincent Hospital, Sydney, Australia; 30 Division of Endocrinology and Metabolism, National Center for Child Health and Development, Tokyo, Japan; 31 Department of Experimental Medicine, Sapienza University of Rome, Roma, Italy; 32 Department of Endocrinology and Internal Medicine, Aarhus University Hospital, Aarhus, Denmark; 33 Université Paris-Saclay, Inserm, Physiologie et Physiopathologie Endocriniennes, Assistance Publique-Hôpitaux de Paris, Hôpital Bicêtre, Service d’Endocrinologie et des Maladies de la Reproduction, Centre de Référence des Maladies Rares de l’Hypophyse, Le Kremlin-Bicêtre, France; 34 Institute of Metabolism and Systems Research, College of Medical and Dental Sciences, University of Birmingham, Birmingham, UK; 35 Centre for Endocrinology, Diabetes and Metabolism, Birmingham Health Partners, Birmingham, UK; 36 Department of Endocrinology, Queen Elizabeth Hospital, University Hospitals Birmingham NHS Foundation Trust, Birmingham, UK; 37 Edison Biotechnology Institute and Heritage College of Osteopathic Medicine, Ohio University, Athens, Ohio, USA; 38 Division of Pediatric Endocrinology and Diabetes, University of California, San Francisco, California, USA; 39 Department of Pediatrics, Tongji Hospital, Tonji Medical College, Hu, China; 40 Department of Endocrinology, St Vincent’s Hospital, Sydney, Australia; 41 Hormones and Cancer Group, Garvan Institute of Medical Research, Sydney, Australia; 42 St Vincent’s Clinical School, Faculty of Medicine, UNSW Sydney, Sydney, Australia; 43 Children’s Healthcare of Atlanta Aflac Cancer and Blood Disorders Service, Department of Pediatrics, Emory University, Atlanta, Georgia, USA; 44 Pituitary Center, Department of Medicine, Cedars-Sinai Medical Center, Los Angeles, California, USA; 45 Divisions of Oncology and Endocrinology, Department of Pediatrics, The Children’s Hospital of Philadelphia, Philadelphia, Pennsylvania, USA; 46 Department of Pediatrics and Pediatric Hematology/Oncology, University Children’s Hospital, Klinikum Oldenburg AöR, Carl von Ossietzki University Oldenburg, Oldenburg, Germany; 47 Department of Medicine, Erasmus University Medical Centre, Rotterdam, the Netherlands; 48 Division of Endocrinology, Health Sciences Graduate Program, Federal University of Sergipe, Aracaju, Sergipe, Brazil; 49 Department of Pediatrics, Osaka University Graduate School of Children, Osaka, Japan; 50 Centro de Investigaciones Endocrinológicas ‘Dr. César Bergadá’, CEDIE-CONICET-FEI, División de Endocrinología, Hospital de Niños Ricardo Gutiérrez, Buenos Aires, Argentina; 51 Medical Faculty, University of Belgrade, Belgrade, Serbia; 52 Department of Pediatrics, Rutgers Robert Wood, Johnson Medical School, New Brunswick, New Jersey, USA; 53 Department of Women’s and Children’s Health, Karolinska Institutet, Stockholm, Sweden; 54 Division of Pediatric Endocrinology, Karolinska University Hospital, Stockholm, Sweden; 55 Department of Endocrinology and Reproductive Medicine, Center for Rare Endocrine and Gynecological Disorders, Pitie Salpetriere Hospital, Sorbonne Université Medecine, Paris, France; 56 Department of Pediatric Endocrinology, Wilhelmina Chilrdren’s Hospital, University Medical Center and Princess Máxima Center for Pediatric Oncology, Utrecht, the Netherlands; 57 Institute of Medicine, Sahlgrenska Academy, University of Gothenburg, Gothenburg, Sweden; 58 Department of Endocrinology, Sahlgrenska University Hospital, Gothenburg, Sweden

## Abstract

Growth hormone (GH) has been used for over 35 years, and its safety and efficacy has been studied extensively. Experimental studies showing the permissive role of GH/insulin-like growth factor 1 (IGF-I) in carcinogenesis have raised concerns regarding the safety of GH replacement in children and adults who have received treatment for cancer and those with intracranial and pituitary tumours. A consensus statement was produced to guide decision-making on GH replacement in children and adult survivors of cancer, in those treated for intracranial and pituitary tumours and in patients with increased cancer risk. With the support of the European Society of Endocrinology, the Growth Hormone Research Society convened a Workshop, where 55 international key opinion leaders representing 10 professional societies were invited to participate. This consensus statement utilized: (1) a critical review paper produced before the Workshop, (2) five plenary talks, (3) evidence-based comments from four breakout groups, and (4) discussions during report-back sessions. Current evidence reviewed from the proceedings from the Workshop does not support an association between GH replacement and primary tumour or cancer recurrence. The effect of GH replacement on secondary neoplasia risk is minor compared to host- and tumour treatment-related factors. There is no evidence for an association between GH replacement and increased mortality from cancer amongst GH-deficient childhood cancer survivors. Patients with pituitary tumour or craniopharyngioma remnants receiving GH replacement do not need to be treated or monitored differently than those not receiving GH. GH replacement might be considered in GH-deficient adult cancer survivors in remission after careful individual risk/benefit analysis. In children with cancer predisposition syndromes, GH treatment is generally contraindicated but may be considered cautiously in select patients.

## Introduction

Survivors of cancer and intracranial tumours may develop growth hormone deficiency (GHD) because of hypothalamic–pituitary dysfunction from the tumour itself, surgical resection, or radiotherapy (1). Due to its key role in promoting linear growth, GH replacement plays an important role in the management of paediatric survivors prior to attainment of adult height (2). Additional benefits of GH on optimizing body composition, bone health, metabolic outcomes, and quality of life provide further rationale supporting the treatment of both children and adults with GHD (3, 4). However, known pro-proliferative, angiogenic, and anti-apoptotic properties of GH and insulin-like growth factor-I (IGF-I) (5) have raised concerns regarding the safety of GH replacement in patients with a history of treatment for cancer or non-malignant tumours and more generally in those at increased risk for malignancy development (6). These concerns primarily stem from* in vitro* and animal model studies and have not been clearly substantiated by clinical observations. GH replacement has been offered to childhood cancer and intracranial tumour survivors in remission from primary disease for many years and has been deemed safe, as outlined in two Endocrine Society practice guidelines (6, 7). However, many areas of uncertainty remain, in particular, regarding individuals who have high cancer or tumour recurrence risks. These include patients who do not achieve complete remission, those with a history of recurrent malignant disease, those on long-term treatment with agents targeting tumour growth, those with a strong family history of cancer, and those with confirmed cancer-predisposing genetic conditions. A diverse international panel of experts was invited by the Growth Hormone Research Society (GRS) to review the evidence pertaining to the safety of GH replacement in survivors of cancer and intracranial tumours and to seek consensus in areas where evidence is conflicting and/or lacking. This report summarizes the proceedings from this Workshop and recommendations agreed upon by the expert panel.

## Methods

The GRS convened a consensus Workshop composed of three virtual sessions on June 9, 16, and September 29, 2021, to review the current state of the field and to address key issues regarding the safety of GH replacement in survivors of cancer and intracranial tumours and in those with cancer predisposition syndromes.

The structure of this Workshop was adapted from prior workshops organized by GRS (8, 9, 10, 11). Due to the COVID-19 pandemic, this Workshop was organized as a virtual meeting that took place on three different occasions. The structure and virtual platform were designed in collaboration with and supported by the European Society of Endocrinology. Fifty-five invited international key opinion leaders from sixteen countries across six continents attended the meeting. These included paediatric and adult endocrinologists with expertise in the management of adults and children with GHD and a history of cancer, paediatric oncologists, epidemiologists, basic scientists, regulatory scientists from the European Medicines Agency and U.S. Food and Drug Administration, and physicians from the pharmaceutical industry. The following societies nominated participants to the Workshop: Chinese Society of Pediatric Endocrinology and Metabolism, Endocrine Society, Endocrine Society of Australia, European Society of Endocrinology, European Society for Paediatric Endocrinology, Japanese Society for Paediatric Endocrinology, Pediatric Endocrine Society, Pituitary Society, Sociedade Brasileira de Endocrinologia e Metabologia and Sociedad Latinoamericana de Endocrinologia Pediatrica.

An extensive critical review based on the current published literature on the safety of GH replacement therapy in cancer and intracranial tumour survivors was written and distributed to all delegates prior to the meeting (12). A planning committee comprising academic adult and paediatric endocrinologists determined the agenda, selected plenary speakers to summarize key relevant topics, and formulated the questions for breakout group discussions.

Five plenary talks summarizing the current state of knowledge were prerecorded and made available to all delegates for review before the first virtual meeting. During the first session, the prerecorded talks were discussed and after pre-defined questions were reviewed, a possible option to revise the questions was afforded for the breakout groups. During the first and second sessions, four breakout groups led by a facilitator and a secretary addressed each topic in greater detail by discussing the list of questions formulated by the planning committee. In order to secure accurate reporting from the breakout groups, all discussion sessions were recorded. All attendees reconvened after each of the breakout sessions to share group reports. At the end of sessions 1 and 2, a writing team compiled the breakout group reports. Another writing team that included the planning committee, facilitators, and secretaries of the breakout groups produced a near final document that was further discussed and reviewed in its entirety and revised by participants during the third and concluding session. When no clear agreement was attained amongst participants, consensus was reached by voting. This draft document was edited further for formatting and references and subsequently circulated only to the academic attendees for final review after the meeting. Participants from pharmaceutical companies were not part of the planning committee or writing team and were not present during text revision on the final day. Scientists from industry were shown the manuscript before submission only to identify possible factual errors. This report is a concise chronicle of the Workshop and is not intended to be an exhaustive review of the literature on this topic. Overall, this consensus statement is derived from: (1) a review paper summarizing current literature produced before the workshop (12), (2) five plenary talks, (3) comments from four breakout groups, and (4) discussion during report-back sessions. The questions asked during the workshop and the key related statements are presented in Table 1.

**Table 1:** The questions and key summary statements from the consensus workshop on safety of growth hormone treatment in survivors of cancer and intracranial tumours.

What is the role of GH-IGF-I in tumour genesis? *In vitro* and* in vivo* models Preclinical data suggest that GH and IGF-I are involved in cancer development. It is not clear how to reconcile the convincing and concerning* in vitro*/*in vivo* data with the reassuring clinical data related to GH replacement and development of cancers. What is the role of GH-IGFI in tumour genesis? Epidemiology Epidemiological studies have shown an association between serum IGF-I levels in the higher normal reference range and an increased risk of certain cancer types, but it is not clear that markedly excessive GH levels in acromegaly are independently associated with increased cancer occurrence. Is GH replacement associated with a higher risk of recurrence of the primary cancer/tumour? Current evidence does not support an association between GH replacement therapy and primary tumour or cancer recurrence in GHD survivors. Is GH replacement associated with a higher risk of a secondary neoplasm? The specific effect of GH replacement on secondary neoplasia risk is minor in comparison to host and tumour treatment-related factors. Is GH replacement associated with a higher risk of death from cancer? Current evidence does not support an association between treatment with GH and increased mortality from cancer among GHD childhood cancer survivors. Should GH replacement be considered in an adult patient previously treated for cancer? GH replacement might be considered in GHD adult cancer survivors (either with childhood- or adult-onset cancer) in remission after careful individual risk/benefit analysis. Should GH replacement therapy be avoided in patients who are in remission from certain malignancies? A decision to prescribe GH replacement therapy in GHD patients with breast, colon, prostate, or liver cancer in remission should be made on a case-by-case basis after detailed counseling about possible risks and benefits and in close conjunction with the treating oncologist. Are there specific considerations related to diagnosing GHD in cancer and intracranial tumour survivors? Specific considerations include the limited reliability of IGF-I levels as a marker for GHD, avoiding the use of GHRH for dynamic testing in patients who have received cranial irradiation, and the need to take into account the presence of other endocrine deficiencies for the interpretation of clinical and laboratory data. Should GH replacement (dosing, serum IGF-I target, monitoring, and transition) be different in patients surviving cancer? GH replacement dosing and monitoring in cancer survivors follow the general recommendations, but closer vigilance is required to avoid over-treatment. How long should providers wait between completion of therapy for cancer or intracranial tumour and the initiation of GH therapy? The timing of initiation of GH therapy following completion of cancer therapy or treatment of an intracranial tumour depends on many factors and should be individualized as a joint decision between treating physicians, patient, and caregivers. This period may be as early as 3 months in children with radiologically proven stable craniopharyngiomas who have significant growth failure and metabolic disturbance and up to at least 5 years in adults with a history of solid tumour such as breast cancer. Are there any specific side effects that may occur after short- and long-term GH replacement? Some side effects related to GH replacement in children occur more frequently among cancer survivors, such as increased intracranial pressure, slipped capital femoral epiphysis, and worsening of scoliosis. In adults, there are no data to suggest a different side-effect profile. Should GH replacement therapy be modified in patients with pituitary tumour or craniopharyngioma after primary surgery? Patients with pituitary tumour or craniopharyngioma remnants receiving GH replacement do not need to be treated or monitored differently than those not receiving GH replacement. Are there special considerations for GH replacement in patients who are on long-term therapy with a tyrosine kinase inhibitor/other chronic therapies for tumour control? For patients with a stable low grade glioma or those on long-term therapy with a tyrosine kinase inhibitor/other chronic therapy, there should be shared decision-making between oncologist, endocrinologist, and the patient/family when considering GH therapy. If cancer occurs in the context of a cancer predisposing genetic condition or strong family history of cancers, should there be additional considerations in starting GH therapy? In children with cancer predisposition syndromes, GH treatment is usually contraindicated but it may be cautiously considered in particular cases with proven GHD. There are no data justifying an absolute contraindication for GH replacement in GH-deficient patients with a strong family history of cancer, so each case needs to be considered individually. Is there a role for long-acting GH (LAGH) preparations in cancer survivors? At this time, there are no data to support LAGH use in cancer survivors.

The key statements in the table should be interpreted in the context of their associated text in the core consensus document.

### Definitions

*Cancer survivor*: An individual is considered a cancer survivor from the time of diagnosis of a malignancy throughout his or her life.

*Primary cancer/neoplasia*: A term used to describe the original or first diagnosed neoplasia.

*Secondary neoplasia*: Refers to a tumour/neoplasm diagnosed after treatment of the primary cancer/neoplasm such as tumours occurring after radiotherapy.

*Cancer predisposition syndrome* also called inherited cancer predisposition, hereditary cancer predisposition, or family cancer predisposition: Genetic mutation(s) that increases the chance of developing cancer at an earlier age compared to the risk for the general population.

*Intracranial tumour survivors*: Survivor after any intracranial tumours including pituitary tumours.

## Background − GH/IGF-I and cancer

### What is the role of GH/IGF-I in tumour genesis? *In vitro* and *in vivo* models

Key statement: Preclinical data suggest that GH/IGF-I is involved in cancer development. It is not clear how to reconcile the convincing and concerning* in vitro*/*in vivo* data with the reassuring clinical data related to GH replacement and development of cancers.

Oncogenic transformation from a normal to a cancerous cell is often accompanied by conferring of growth factor autonomy (13, 14). The ligand receptor pairs of GH/GH receptor (GHR) and IGF-I/IGF-I receptor (IGF-IR), although not proto-oncogenes or oncogenes themselves, frequently form an autocrine/paracrine loop implicated in multiple facets of cancer physiology (5, 15, 16). It has been suggested that GH and IGF-I are permissive, but not causative, for malignant growth (17). In normal tissues, GH action induces IGF-I production, and the two hormones have mutually overlapping and exclusive effects on tissues expressing the respective cognate receptors. In cancer development, the GH-IGF axis, IGF-II, insulin receptors, and hybrid receptors have important roles in the tumour and the tumour microenvironment driving pro-proliferative, angiogenic, and anti-apoptotic signalling for tumour cell survival and growth (5). Moreover, autocrine GH-IGF act to impact cancer resistance against various therapies and to initiate the metastatic process of epithelial-to-mesenchymal transition and induction of cancer stem cell niches (18). The combined effect of GH-IGF action could potentially contribute to tumour development, metastases, and relapse.

Many types of human cancers express GH, GHR, IGF-I, and/or IGF-IRs in the tumour or the tumour microenvironment, thus providing an opportunity for GH/IGF-I to act in an endocrine, paracrine, or autocrine manner. Despite age-dependent reduction in the production of pituitary (endocrine) GH and, therefore, IGF-I, extra-pituitary/local production of these growth factors is often responsible for supporting an oncogenic niche (19, 20). Several recent studies have described a pattern of GH supported ‘field cancerization’ promoting conditions for oncogenic transformation, proliferation, malignancy, and relapse (20). For example, DNA damage after age-associated mutations or external insults leads to a p53-dependent increase in local GH production. This increase in GH then leads to suppression of p53 expression, thereby diverting the cellular commitment to survival and proliferation (17, 21). Additionally, increased GH action in normal and tumour tissues suppresses DNA damage repair, enabling an increase in the cellular mutational burden and facilitating the onset of dysplasia (22). These observations explain the intracellular mechanism(s) dictating the various cancer phenotypes.

Additional support for the importance of GH in cancer progression is the finding that GH-induced intracellular signalling pathways have been identified as the third most highly associated pathway among 421 pathways with breast cancer susceptibility containing 3962 genes in a human genome-wide association study (23). Therefore, establishing molecular connections between how and where GH and/or IGF-I action originates and their influence in cancer properties is important when developing therapeutic strategies.

Multiple mouse models of GH resistance and deficiency closely reinforce the hypothesis that a lack of GH action may provide an ‘onco-protective’ phenotype (24). For example, the GHR−/− mouse (the Laron mouse) is GH resistant with low IGF-I and high GH levels. These mice are resistant to diet-induced diabetes and cancer, paralleling the phenotype of patients with Laron syndrome (24, 25). Also, rats with GHD are resistant to chemically induced mammary carcinogenesis but can be made susceptible by administering GH; once mammary cancers are established, halting GH administration causes the cancers to regress (26). Recently, inhibition of GH action has been found to overcome chemotherapy resistance* in vitro* (27) in mice (28, 29) and rats (30).

Although several studies over the last 25 years have suggested that the GH-IGF axis is a potential therapeutic target in cancer leading to development of peptides, antibodies, and small molecules aimed at inhibiting their action, these agents have not been proven to be effective in clinical trials (15). In light of this current knowledge of the role of GH/IGF-I in cancer development, it is pertinent to reflect upon the feasibility and safety of using GH replacement in cancer survivors.

### What is the role of GH-IGF-I in tumour genesis? Epidemiology

Key statement: Epidemiological studies have shown an association between serum IGF-I levels in the higher normal reference range and an increased risk of certain cancer types, but it is not clear that markedly excessive GH levels in acromegaly are independently associated with increased cancer occurrence.

Several epidemiological studies and systematic reviews with meta-analyses have drawn attention to a possible association between serum IGF-I levels in the higher normal reference range with the presence of breast, colorectal, and prostate cancer in the general population (31, 32, 33, 34, 35). Nevertheless, a causal relationship is difficult to determine from these studies due to the presence of multiple confounders, such as age, body weight and height, nutritional status, insulin resistance, heterogeneity of IGF-I assays, and serum IGFBP3 levels (36, 37, 38, 39, 40). In addition, it has not been possible to translate results of epidemiological studies into clinical practice in order to establish a ‘safe’ IGF-I level.

Perhaps the most significant* in vivo* human observations implicating GH/IGF-I in cancer is the case of patients with Laron syndrome. Generally, these patients possess homozygous inactivating mutations of the GH receptor gene (GHR−/−), and thus, they are GH-resistant resulting in short stature with very low IGF-I and high GH levels. Although these patients have increased adiposity, no cancer has been found in an Ecuadorian cohort of patients with Laron syndrome, in contrast with cancer rates of >20% in heterozygous (GHR+/−) relatives and the control population (25, 41). Similarly, low malignancy risk has been reported in an Israeli Laron syndrome cohort (42, 43). Cancer rates are also low but not completely absent in a Brazilian cohort of individuals with isolated congenital GHD due to a GHRH-receptor gene mutation, who have very low, but detectable, serum levels of GH and IGF-I (44).

The relationship between serum levels of GH and IGF-I with increased risk of cancer in acromegaly has been long debated (45, 46). While there are studies demonstrating an increased risk of colorectal and thyroid cancer, other studies do not find this association (45, 46). More recent nation-wide studies including unselected cohorts of patients with acromegaly have shown an increased risk of malignancy but no increased cancer mortality (47, 48). These conflicting findings might be explained by variations in biochemical control with treatment and/or the presence of other factors unrelated to GH/IGF-I excess, such as age, insulin resistance, hyperinsulinemia and diabetes, and surveillance bias, as well as methodological differences (49). More recent studies have shown that mortality in acromegaly patients is normalized with biochemical control of the disease, resulting in increased life expectancy and death due to cancer to a level observed in the general population (50).

## Major safety issues with GH replacement of cancer and intracranial tumour survivors during childhood and adulthood

### Is GH replacement associated with a higher risk of recurrence of the primary cancer/tumour?

Key statement: Current evidence does not support an association between GH replacement therapy and primary tumour or cancer recurrence in GHD survivors.

The evidence is based on several studies of childhood cancer survivors who did or did not receive GH replacement (51, 52, 53, 54, 55, 56, 57, 58, 59, 60), including a meta-analysis from the Endocrine Society (61). Data pertaining to the risk for cancer recurrence in survivors treated with GH during adulthood are limited, but more robust data have been produced for adult patients with benign pituitary adenomas and craniopharyngioma. These data do not support an association between GH replacement therapy and tumour recurrence. The studies are, however, limited by small numbers of participants (62), their focus on nonmalignant pituitary tumours (63, 64), selection bias, and relatively short follow-up durations (65).

While the evidence concerning GH replacement and risk of cancer/tumour recurrence is generally reassuring, it is important to acknowledge the limitations of studies reporting on tumour outcomes in survivors treated with GH. These include reliance on self-reported data (52, 54), retrospective design with potential selection bias (the likely prescription of GH to patients with the lowest cancer recurrence risk) (61), and the lack of adjustment for additional variables, such as time elapsed between cancer remission and initiation of GH therapy (6). Furthermore, safety data from historical cohorts of survivors may not be applicable to patients treated under newer protocols, such as those utilizing targeted chemotherapy with tyrosine kinase inhibitors for persistent disease (66, 67). In addition, GH treatment protocols vary across time and region. Moreover, there is a paucity of data related to rare tumours involving the hypothalamic–pituitary area, such as chordoma, pituicytoma, optic gliomas, and germinomas.

Existing practice guidelines have not specifically addressed GH management in survivors who experience tumour recurrence while receiving GH replacement, as long-term outcomes pertaining on these patients are lacking (2, 3, 6, 7, 68). The panel agreed that GH replacement should be discontinued when disease relapse or clinically significant tumour progression is confirmed (3, 7). Shared decision making between the endocrinologist, the patient, caregivers when applicable, and the oncologist should make an individualized plan regarding the resumption of GH therapy after tumour remission is reachieved. The panel agreed that there is insufficient evidence to guide recommendations as to when GH replacement can resume after remission. However, drawing from experience with the treatment of the primary disease, it is of the panel’s opinion that in paediatrics, resumption of GH replacement could be considered 1 year after remission from cancer relapse. A shorter time period may be acceptable for non-malignant tumours and craniopharyngioma, but there is a need for additional data in this area (7). In adults, relapsed malignant disease is a contraindication for GH treatment, and this therapy can only be resumed when the malignancy is considered cured. Given that GH replacement has not been shown to influence tumour progression (recurrence and/or growth) of pituitary adenomas or craniopharyngioma in adults, an individualized shared decision should be made regarding the resumption of GH therapy based on factors such as the underlying diagnosis, degree of tumour progression, and the extent of the intervention that was required to achieve relapse (68).

### Is GH replacement associated with a higher risk of a secondary neoplasm?

Key statement: The specific effect of GH replacement on secondary neoplasia risk is minor in comparison to host- and tumour treatment-related factors.

A significant association between GH replacement and a higher risk for secondary neoplasia has been reported in some (52, 54), but not all (55, 61, 69, 70, 71), studies investigating health outcomes in childhood cancer survivors, as well as a meta-analysis from the Endocrine Society (61). Host- and treatment-related factors, such as radiotherapy, are the primary drivers of secondary neoplasia risk in this population (72, 73).

Multiple contributing host (age, sex, genetic predisposition), tumour (type and latency period), and treatment (organ irradiation, chemotherapy) risk factors for subsequent neoplasia complicate determining whether there is a specific risk from GH replacement (72, 74). Radiotherapy, in particular, is a known major risk factor for both GHD (when radiation fields involve the hypothalamic–pituitary region) (1) and secondary neoplasia affecting radiation-exposed areas (73). It is therefore challenging to identify a specific contribution of GH replacement beyond the risk already posed by radiotherapy. The increased risk for secondary neoplasms among individuals treated with GH in reports from the Childhood Cancer Survivor Study was primarily driven by a higher than expected occurrence of meningioma, a tumour known to occur after CNS irradiation (52, 54). These results were based on a relatively small number of events, and the possibility of ascertainment bias could not be excluded. Subsequent studies focusing on secondary CNS neoplasia (69) and more specifically meningioma (70, 71, 75) did not support a significant contribution of GH to the substantial risk already conferred by cranial radiotherapy. A more recent report from the SAGhE cohort showed no significant associations between the duration of and the dose used for GH replacement and the occurrence of meningioma (75). While these results are generally reassuring, longer term studies are still needed to fully understand whether this risk may be modified, or secondary tumour growth accelerated, by treatment with GH (71, 76) particularly given the long latency and frequently asymptomatic nature of radiation-induced meningioma (77). Treatment with GH should not alter the surveillance plan for survivors at risk for secondary CNS neoplasm, as discussed below (71).

Existing practice guidelines have not specifically addressed GH management in survivors who experience secondary neoplasia while on GH, as long-term outcome data pertaining to these patients are lacking (2, 3, 6, 7, 68). Although available evidence does not support a strong association between GH replacement and secondary neoplasia, the panel agreed that GH should be discontinued when a secondary neoplasm is diagnosed and that shared decision-making between the endocrinologist, the patient, their family when applicable, and the oncologist should make an individualized decision regarding resumption of GH after remission from the secondary neoplasia is achieved. Drawing from experience with the management of primary disease, the panel agreed that in paediatrics, resumption of GH replacement could be considered 1 year after remission from a secondary neoplasm (7). Meningiomas are the most frequently reported secondary neoplasm in patients who have been treated with GH; the risk for meningioma seems to be primarily related to cranial radiation which independently causes both meningioma development (73, 78) and GHD. In a recent report, the risk of developing meningioma in patients with childhood-onset GHD was not associated with age at first GH treatment, mean daily dose, duration of treatment, or cumulative doses (75). Hence, GH replacement *per se* does not appear to confer additional risk to the development of meningioma and the group concurred that individuals with stable meningiomas and GHD could be treated with GH. However, given the somewhat discordant results on the association between GH replacement and the risk for meningioma (52, 54, 55, 69), an individualized decision should thus be made for affected patients on whether to resume GH and the timing of restarting GH after close communication with the patient and the oncologist.

### Is GH replacement associated with a higher risk of death from cancer?

Key statement: Current evidence does not support an association between treatment with GH and increased mortality from cancer among GHD childhood cancer survivors.

The lack of association between GH replacement and cancer mortality has been reported in several retrospective studies of childhood cancer survivors (52, 54, 79). A multicenter European study showed (a) no increase of morbidity and mortality from cancer in a low-risk group including isolated GHD, idiopathic short stature and small for gestational age; (b) increased incidence of bladder and bone tumours in the intermediate-risk group including multiple pituitary hormone deficiencies and syndromes; (c) increased morbidity and mortality from almost all types of tumours in the high-risk group including patients with previous history of cancer (80). Nevertheless, these results were based on a small number of events, and the studies lacked non-GH treated cancer survivor controls, a significant limitation given the known risks for subsequent neoplasia due to a variety of host and cancer treatment factors (72). A more recent analysis of this study did show that overall mortality was associated with the underlying condition and not the mean daily or cumulative doses of GH (79). Existing data have additional limitations, including reliance on self-reporting for GH treatment results (52, 54), retrospective design with possible selection bias (prescription of GH to patients with the lowest mortality risk from cancer), and short follow-up durations, especially among survivors treated with more recent regimens. While associations between GH replacement and excess mortality from cancer will require continued assessment, the adverse impact of untreated GHD in these patients should also be considered (81). Whether untreated GHD contributes to all-cause mortality and if its consequences could be mitigated by measures other than GH replacement in ageing cancer survivors remain areas for further research (1).

## GH replacement in adult survivors of cancer and intracranial tumours

### Should GH replacement be considered in an adult patient previously treated for cancer?

Key Statement: GH replacement might be considered in GHD adult cancer survivors (either with childhood- or adult-onset cancer) in remission after careful individual risk/benefit analysis.

In the absence of data unequivocally linking GH replacement with cancer relapse or the development of a secondary neoplasm in GHD adult cancer survivors, the potential benefits of therapy on health outcomes allow for considering GH replacement in adults with a history of cancer in remission. However, long-term safety data remains limited, and these are derived from voluntary surveillance registries and not from long-term, prospective, controlled trials. Therefore, this recommendation is primarily based on expert opinion (12). Additional caution should be applied in the counselling of survivors of cancers diagnosed during adulthood given the paucity of data pertaining to the safety of GH in this population (in contrast to childhood cancer survivors) and differences in the most prevalent types of cancer between children (leukaemia and CNS malignancies) and adults (breast, prostate, and colon). Adult-onset GHD may be underdiagnosed due to its relatively non-specific symptoms (17) and possibly because of limited access to specialized care and/or dynamic GH testing (81). These factors further challenge our understanding of the true impact of GHD on adult cancer survivors and consequently of the benefit of GH replacement in this specific population.

### Should GH replacement therapy be avoided in patients who are in remission from certain malignancies?

Key Statement: A decision to prescribe GH replacement therapy in GHD patients with breast, colon, prostate, or liver cancer in remission should be made on a case-by-case basis after detailed counselling about possible risks and benefits and in close conjunction with the treating oncologist.

Although there are no clinical data to inform this recommendation, when the role of GH-induced intracellular signalling pathways is considered, and given data derived from* in vitro* as well as animal models, initiation of GH replacement in patients with a history of some solid tumours should be made with caution (23). These include breast cancer, hepatocellular carcinoma (where GH receptor expression may be high), prostate cancer, and colorectal cancer (34). In the absence of safety data, it was the consensus of the panel that it would be reasonable to delay the onset of GH replacement until patients with these conditions are in remission for 5 or more years (see further the section ‘How long should providers wait between completion of therapy for cancer or intracranial tumour and the initiation of GH therapy’?).

## Diagnostic testing and GH therapy in survivors of malignancies

### Are there specific considerations related to diagnosing GHD in cancer and intracranial tumour survivors?

Key statement: Specific considerations include the limited reliability of IGF-I levels as a marker for GHD, avoiding the use of GHRH for dynamic testing in patients who have received cranial irradiation, and the need to take into account the presence of other endocrine deficiencies for the interpretation of clinical and laboratory data.

#### Whom and when to test

In patients with a clinical suspicion of GHD, the presence of risk factors such as a sellar/suprasellar tumour, hypothalamic–pituitary surgery, hypothalamic/pituitary radiation dose of  ≥ 18 Gy, a single fraction total body irradiation ≥ 10 Gy or fractionated ≥ 12 Gy, younger age at cancer/tumour treatment, longer elapsed time since treatment, or a low IGF-I level may guide the choice of testing and later re-testing (7).

A normal serum IGF-I level does not exclude the presence of GHD. Children with serum IGF-I levels <0 s.d. should be evaluated further. Additionally, those with IGF-I levels >0 s.d. but with a high pre-test probability of GHD (e.g. high dose hypothalamic/pituitary radiation or multiple pituitary hormone deficits) should also be evaluated (7, 82). Patients with risk factors precluding testing or with intermediate GH peak levels should be followed clinically. Patients at a significant risk for developing GHD over time (i.e. irradiated patients) should have ongoing evaluation of the GH axis, particularly if clinical signs of GHD are present such as failing growth in children (7, 83). However, controversies continue regarding optimal protocols including the timing and frequency of testing.

In those with childhood-onset GHD, the GH-IGF-I axis should be reevaluated in patients during the transition, except in those with multiple pituitary hormone deficiencies due to hypothalamic tumours, previous high dose radiotherapy (>30 Gy), or hypothalamic–pituitary surgery (84, 85). In the latter patients, GH replacement need not be discontinued. GHD should be confirmed in all other patients with childhood-onset GHD by re-testing after patients have reached their near-adult height (a height velocity of less than 1–2 cm/year or by bone age confirmation) (86). The recommended time between discontinuing GH therapy and re-testing is 1 month (87, 88, 89).

Other hormonal deficits, especially for thyroid hormone, cortisol, and sex-steroids must be investigated and optimally treated before provocative testing for GH is performed.

#### Which tests and what cut-off values confirm the diagnosis of GHD?

Insulin-induced hypoglycemia during an insulin tolerance test (ITT) is considered to be the ‘gold standard’ for the diagnosis of GHD (90, 91). The diagnostic criteria based on peak GH in the ITT for adult GHD, childhood GHD, and transition age (final height to peak bone mass) do not differ between those surviving cancer and/or intracranial tumour and those with GHD from other causes (2, 7, 83, 87, 92, 93, 94, 95, 96, 97, 98, 99). There are a number of contraindications to ITT, however, that are of particular relevance in cancer and intracranial tumour survivors that may limit its utility, including patients with a history of seizures or ischemic heart disease, when the ITT should generally be avoided.

Alternative protocols for provocative stimulation of GH include glucagon, clonidine, arginine, or arginine with growth hormone-releasing hormone (GHRH) (100). The use of GHRH alone or with arginine to diagnose GHD in cancer survivors with sellar or parasellar tumours after surgery or radiation is not recommended, as the GH response may be falsely normal (7, 83). Glucagon (GST) and/or arginine stimulation (AST) tests are therefore the most frequently used alternatives, although both have well described limitations in this population (7, 98). Of particular consideration are the impact of irradiation on the reliability of the AST in children and adults (101), the impact of overweight/obesity on the cut-offs for diagnosis in the GST in adults (98, 102), and sex steroid hormone status.

Macimorelin is an oral GH secretagogue that has been validated as a diagnostic test for adults with GHD but not yet in children and not specifically in the setting of cancer or intracranial tumour survivors who have received irradiation to the hypothalamus and/or pituitary (103). There was consensus that the advantage of an oral preparation with minimal side effects is appealing in this population but that relevant studies to determine the GH cut-points are required before it can be routinely recommended in children and in those patients who have received irradiation to the hypothalamus and/or pituitary.

### Should GH replacement (dosing, serum IGF-I target, monitoring, and transition) be different in patients surviving cancer?

Key statement: GH replacement dosing and monitoring in cancer survivors follow general recommendations, but a higher degree of vigilance is required to avoid over-treatment.

There are no data to support management recommendations for cancer survivors that differ from those available for other populations of patients with GHD (7). In children, monitoring height velocity is central, and an acceptable growth response can be achieved in most children with a low starting dose of GH followed by slow dose up-titration (3, 7). Serum IGF-I is an important safety marker during GH replacement in childhood and adult cancer survivors and should be measured after making a dose adjustment, approximately every 3 months during dose titration and at least annually thereafter. Targeting a serum IGF-I level within the normal range whilst optimizing growth is recommended. Headache may be a symptom of GH overdosing in children, and in adults, pedal oedema and arthralgias may be experienced when excessive GH doses are administered (2). The growth response to a given GH dose may be reduced in children exposed to spinal radiation and in children receiving pharmacological glucocorticoid therapy (12).

The management of GH replacement in childhood cancer survivors during the transition period does not differ from that of other childhood-onset GHD patients (2). The dose is often reduced in the transition period, although a higher GH dose is typically needed with the onset of oral oestradiol treatment to maintain the serum IGF-I level (3).

In adults, serum IGF-I is also an important safety biomarker and should be maintained within the normal range (7). No single efficacy marker is available for adult GH replacement, and health-related quality of life might be challenged in cancer survivors for many reasons other than GHD.

Childhood and young adult cancer survivors and their medical team should be aware of the risk of subsequent CNS neoplasms in patients who have undergone cranial irradiation. There is no evidence that GH increases this risk, and therefore, decisions with regard to timing of MRI scans should follow standard practice and recent guidelines (104).

### How long should providers wait between completion of therapy for cancer or intracranial tumour and the initiation of GH therapy?

Key statement: The timing of initiation of GH therapy following completion of cancer therapy or treatment of an intracranial tumour depends on many factors and should be individualized as a joint decision between treating physicians, patient, and caregivers. This period may be as early as 3 months in children with radiologically proven stable craniopharyngiomas who have significant growth failure and metabolic disturbances and up to at least 5 years in adults with a history of a solid tumour such as breast cancer.

There are no data to guide when to initiate GH replacement after the completion of primary tumour therapy and whether this timing affects disease recurrence. The timing of initiation of GH replacement should be individualized and carefully reviewed with the patient, family, and oncologist or neurosurgeon. The clinical status of the patient, the type of tumour (malignant vs non-malignant (pituitary adenoma/craniopharyngioma)), and treatment modality are important factors in this decision (7). In children, initiation of GH should be considered when declining height velocity is detected and GHD is biochemically confirmed (2).

In children with craniopharyngioma and radiologically stable disease, testing for GHD and commencement of GH therapy as early as 3 months post treatment is reasonable for improving growth and body composition in some children. For other types of tumours, it is advisable to wait at least 1 year following the end of tumour treatment and only when radiologically confirmed stability is achieved, considering that tumour relapse is highest during the first 12 months after cancer treatment (7).

For adults with craniopharyngioma and pituitary adenomas, a waiting period of 12 months was discussed in order to secure adequate evaluation of GHD treatment and diagnosis, but consensus was not reached on this and some experts considered it safe to start GH replacement after pituitary surgery for craniopharyngiomas or for benign pituitary adenomas without a waiting period as long as other pituitary hormone deficiencies are adequately replaced. For other adult onset cancers for example, breast cancer, we recommend at least a 5-year disease-free interval before commencement of GH replacement therapy.

### Are there any specific side effects that may occur after short- and long-term GH replacement?

Key statement: Some side effects related to GH replacement in children occur more frequently among cancer survivors, such as increased intracranial pressure, slipped capital femoral epiphysis, and worsening of scoliosis. In adults, there are no data to suggest a different side-effect profile.

After starting GH replacement, re-testing for central hypothyroidism and adrenal insufficiency may be needed, and in those already on treatment for these deficiencies, dose adjustment may be required as reviewed and stated in previous guidelines (2, 7).

There are no data in adults to indicate that side effects of GH replacement differ from those seen in patients without a history of cancer. In children, however, increased intracranial pressure, slipped capital femoral epiphysis, and worsening of scoliosis may be more frequent among cancer survivors (7). In children who have received spinal irradiation, disproportionate growth may be exaggerated with GH therapy, as the spine may grow proportionally less than the limbs (105).

### Should GH replacement therapy be modified in patients with pituitary tumour or craniopharyngioma after primary surgery?

Key statement: Patients with pituitary tumour or craniopharyngioma remnants receiving GH replacement do not need to be treated or monitored differently than those not receiving GH replacement.

Craniopharyngiomas express GH receptors (106), and increased GH receptor abundance may be associated with tumour aggressiveness (107). When exogenous GH is added to craniopharyngioma cells* in vitro*, cell growth occurs (108). However,* in vivo* case control studies of children and adults with craniophiopharyngiomas (109, 110, 111) and non-functioning pituitary adenomas (112, 113) show no increased risk of recurrence or tumour progression with GH therapy, including in those patients who have post-operative tumour remnants and in those patients treated with or without radiotherapy. Pharmaceutical company-sponsored post-marketing surveillance studies show similar findings (63, 114, 115, 116, 117). Thus, there was an agreement among Workshop delegates that there is no current evidence to suggest that there should be a difference in treating or monitoring patients with pituitary tumour remnant after primary surgery who are receiving GH replacement or not. If these tumours were to recur, the consensus would be to consider discontinuation of GH and revisiting the possibility of re-introducing GH at a later date taking into considerations specific tumour and patient characteristics.

### Are there special considerations for GH replacement in patients who are on long-term therapy with a tyrosine kinase inhibitor/other chronic therapies for tumour control?

Key statement: For patients with a stable low-grade glioma or those on long-term therapy with a tyrosine kinase inhibitor/other chronic therapy, there should be shared decision-making between oncologist, endocrinologist, and the patient/caregiver when considering GH therapy.

There is an increasing number of patients whose disease is controlled with chronic use of tyrosine kinase inhibitors (TKIs) or other targeted chemotherapies. Of concern is the overlap between the tyrosine kinase pathways being targeted and the cellular pathways for GH axis signalling (7). Currently, there are no data to support safety or harm from GH therapy for patients receiving TKI (or other) therapy or after completion of such therapy. Given that a significant deterioration in linear growth might occur during the prolonged treatment course in children, the group felt it reasonable to consider GH therapy in these individuals with confirmed GHD, after consultation with the oncologist and after informed discussion with the patient and parents/guardians. However, the group advocated for developing a platform to collect data and to report adverse events. It was noted that some children may have neurofibromatosis-I, and in these patients, low-grade tumours may progress with or without GH therapy. The risk/benefit ratio of GH therapy in adults with stable disease while receiving TKIs/other therapies is currently not clearly defined.

### If cancer occurs in the context of a cancer predisposing genetic condition or strong family history of cancers, should there be additional considerations in starting GH therapy?

Key statement: In children with cancer predisposition syndromes, GH treatment is usually contraindicated but it may be cautiously considered in particular cases with proven GHD. There are no data justifying an absolute contraindication for GH replacement in GH-deficient patients with a strong family history of cancer; each case needs to be considered individually.

Individuals with a cancer predisposition syndrome have a genetic mutation that increases their risk for developing cancer compared to the general population. Most have mutations in genes encoding tumour suppressors that would normally sense DNA damage and promote DNA repair (118) for example, Bloom syndrome, Li Fraumeni syndrome, Lynch syndrome, and Fanconi anemia. As GH and IGF-I may reduce the time for DNA repair (119), there is concern about the use of GH in these individuals, and in general, it was deemed to be contraindicated. However, there is a subset of children with GHD who have not developed a malignancy in whom GH therapy may be considered. For example, patients with Fanconi anemia may present with GHD and have ectopic posterior pituitary and/or pituitary stalk interruption syndrome (120), and the patient and parents/guardians may need to weigh the potential cancer risks vs extreme short stature in adulthood.

Activating mutations in growth-promoting oncogenes, including those encoding tyrosine kinase receptors and other intracellular signalling proteins (118), for example, RASopathies, constitute a group of rare conditions involving in the Ras/MAPK cell signalling pathway, such as Noonan syndrome, neurofibromatosis-I, Costello syndrome, and Legius syndrome. There is no evidence suggesting increased risk of GH therapy on development of malignancy in patients with Noonan syndrome who do not have a prior malignancy (121) or in patients with neurofibromatosis-I who are treated with GH therapy for short stature, but patient numbers are small, and longer-term data are needed (80, 122, 123, 124). In this context, it should be acknowledged that overlapping clinical features among genetic syndromes associated with increased cancer risk and late appearance of their hallmark features make the diagnosis challenging for many of these syndromic patients (124).

The consensus was that GH replacement could be cautiously considered in children with a RASopathy and confirmed GHD after informed discussion with the patient and parents/guardians. There are no data regarding GH replacement in adults with cancer predisposition syndromes, and the consensus of the group was that these patients should not receive GH.

A high degree of caution is needed when treatment is considered in individuals with familial cancers such as familial adenomatous polyposis and BRCA 1/2-mutation positive breast cancer, or with underlying cancer predisposition syndromes, such as the multiple endocrine neoplasia syndromes. These considerations may also apply to patients with a history or a strong family history of breast, colon, and prostate cancers. Notably, patients who have received prior radiotherapy to the breast or lungs (for example chest/mediastinal/mantle radiotherapy) are also at increased risk of developing second malignancies, a risk that is further increased in those with a genetic susceptibility to such tumours (80, 125). To date, there are no data justifying an absolute contraindication for GH therapy in these patients, so each patient needs to be considered individually, as lack of data should not automatically exclude GHD patients from GH replacement (12).

However, only a minority of patients undergo routine testing for cancer predisposition syndromes (123, 124, 126). It may be prudent to consider the potential benefits of background genetic screening in some patients with a family history of cancer prior to the initiation of GH replacement, recognizing the potential harm that such screening may entail.

### Is there a role for Long-Acting GH (LAGH) preparations in cancer survivors?

Key statement: At this time, there are no data regarding LAGH use in cancer survivors.

LAGH preparations have different pharmacokinetics and pharmacodynamics than daily GH. While mean IGF-I is comparable between daily and weekly GH replacement, levels are higher immediately after an injection and lower immediately before a subsequent injection in patients receiving LAGH (8, 127). Given that currently there are very limited data, the group was in consensus that data should be prospectively collected in non-childhood cancer survivors first, and if no safety concerns are observed, then studies in childhood cancer survivors should be undertaken to explore this question further.

## Conclusions

During the time period of the Workshop, 15 key summary statements were produced with a strong consensus among the participants. The decision to test for GHD and replace GH in children and adults who have survived cancer and those with a high genetic susceptibility to develop cancer can be challenging and should only be considered if these patients had a suggestive history of possible GHD such as structural hypothalamic/pituitary disease, surgery or irradiation in these areas, head trauma, or evidence of other pituitary hormone deficiencies, in line with previous clinical practice guidelines (3). This consensus document has been generated to support physicians, patients, and their families in this decision-making. The document will also guide individual decisions regarding initiation of GH replacement in patients with cancer and intracranial (including pituitary) tumours.

## Declaration of interest

M C S B: lecture fees and/or consultancy honoraria from Merck, Pfizer, Sandoz, Novo Nordisk. L E C: lecture and/or consultancy honoraria from Novo Nordisk and Sandoz, Pfizer site PI on long-acting growth hormone study and sub-PI on investigator-initiated research grant to Boston Children’s Hospital. A R H: consultant for Novo Nordisk and Ascendis. M P: Research support from Pfizer, Novo Nordisk, IPSEN, Sandoz, Merck-Serono, Lilly, Sanofi, National French Grants, public funding, ANR and PHRC, and Scientific advisory board: Increlex (IPSEN), GNAP (Novo Nordisk), KIGS France (Pfizer). K C J Y: Research grants to Barrow Neurological Institute from Ascendis, Crinetics, Amryt, and Corcept, and Scientific Advisory Boards for Novo Nordisk, Ascendis, Amryt, Ipsen, Strongbridge, Recordati, and Corcept. M B: Research support, lecture fees, and/or consultancy honoraria from Diasorin, Genexine, Genescience, IDS, IPSEN, Merck, Novartis, OPKO, Pfizer, Roche, Sandoz. B M K B: PI of research grants to Massachusetts General Hospital from Ascendis, Crinetics and Ionis and occasional consultant for Novo Nordisk, HRA Pharma, Merck Serono, Ipsen, and Recordati. C S Y C: Investigator on OPKO-sponsored Long-Acting Growth Hormone study. S C: Consultant for Novo Nordisk and Sandoz. P E C: Research grant from Novo Nordisk Chair, Data Monitoring Committee for the Ipsen Increlex Registry; Member of the Scientific Advisory Board for Lumos Pharma. R C: Scientific advisory board Pfizer, Novo Nordisk and Merck-Serono. A F: Scientific advisory board: Pfizer, Novo Nordisk, Merck-Serono, Research support: Novo Nordisk, Sandoz. A G: The 2020 Growth Hormone Research Competitive Grant Program Award from Pfizer. K K Y H: Advisory Board, Novo Nordisk. R H: Advisory board, research grant and lecturer: Novo Nordisk, Pfizer, Opko, Ascendis, Sandoz. A M I: Consultancies for Recordati, Serono Fundation, Novo Nordisk Foundation, Ipsen and grants from Takeda and Pfizer. J O L J: Consulting fee from Novo Nordisk and unrestricted research grants from Pfizer. P K: Invitations to congresses and lecturing fees from Pfizer and Ipsen. N K: Research and educational grants, advisory board and lecturing fees from Pfizer. S M: Research grant to Institution from Pfizer and Consultant for Novo Nordisk. H M: has received reimbursement of participation fees for scientific meetings and continuing medical education events from Ferring, Lilly, Pfizer, Sandoz/Hexal, Novo Nordisk, Ipsen, and Merck Serono. Reimbursement of travel expenses from Ipsen and lecture honoraria from Pfizer. Supported by the German Childhood Cancer Foundation, Bonn, Germany. L S: Personal fees and non-financial support from Ascendis, Hexal, Merck, Novo Nordisk, Pfizer, and Sandoz. P T: Lectures fees from Novo Nordisk, Ipsen, Pfizer, Merck. G J: Consulting fee from Novo Nordisk and unrestricted research grants from NovoNordisk and Pfizer.

## Funding

The consensus workshop was funded by the Growth Hormone Research Society with a non-financial support from the European Society of Endocrinology http://dx.doi.org/10.13039/100010382.
